# Effective Alu Repeat Based RT-Qpcr Normalization in Cancer Cell Perturbation Experiments

**DOI:** 10.1371/journal.pone.0071776

**Published:** 2013-08-14

**Authors:** Ali Rihani, Tom Van Maerken, Filip Pattyn, Gert Van Peer, Anneleen Beckers, Sara De Brouwer, Candy Kumps, Evelien Mets, Joni Van der Meulen, Pieter Rondou, Carina Leonelli, Pieter Mestdagh, Frank Speleman, Jo Vandesompele

**Affiliations:** Center for Medical Genetics, Ghent University Hospital, Ghent, Belgium; Deutsches Krebsforschungszentrum, Germany

## Abstract

**Background:**

Measuring messenger RNA (mRNA) levels using the reverse transcription quantitative polymerase chain reaction (RT-qPCR) is common practice in many laboratories. A specific set of mRNAs as internal control reference genes is considered as the preferred strategy to normalize RT-qPCR data. Proper selection of reference genes is a critical issue, especially in cancer cells that are subjected to different *in vitro* manipulations. These manipulations may result in dramatic alterations in gene expression levels, even of assumed reference genes. In this study, we evaluated the expression levels of 11 commonly used reference genes as internal controls for normalization of 19 experiments that include neuroblastoma, T-ALL, melanoma, breast cancer, non small cell lung cancer (NSCL), acute myeloid leukemia (AML), prostate cancer, colorectal cancer, and cervical cancer cell lines subjected to various perturbations.

**Results:**

The geNorm algorithm in the software package qbase+ was used to rank the candidate reference genes according to their expression stability. We observed that the stability of most of the candidate reference genes varies greatly in perturbation experiments. Expressed Alu repeats show relatively stable expression regardless of experimental condition. These Alu repeats are ranked among the best reference assays in all perturbation experiments and display acceptable average expression stability values (M<0.5).

**Conclusions:**

We propose the use of Alu repeats as a reference assay when performing cancer cell perturbation experiments.

## Background

Reverse transcription quantitative polymerase chain reaction (RT-qPCR) has proven to be a reliable method to quantify gene expression. Correct normalization is a critical issue for accurate interpretation of RT-qPCR results. This can be achieved using several strategies such as ensuring similar numbers of cells, similar amounts of input RNA, applying internal control reference genes like ribosomal RNAs (rRNAs) or messenger RNAs (mRNAs), or merging multiple strategies in one protocol [1], [2].

The use of mRNAs as internal control reference genes for normalizing RT-qPCR data is being applied widely [2]–[6]. However, this strategy should be carried out carefully as its accuracy depends directly on the expression stability of the selected reference genes. According to the Minimum Information for Publication of Quantitative Real-Time PCR Experiments (MIQE guidelines) [7], it is no longer accepted to consider that certain reference genes are stable by convention. Our group has previously reported a strategy for accurate normalization of RT-qPCR data based on geometric averaging of multiple stably expressed internal control genes [4]. In this study, we show that the choice of reliable internal controls is of particular importance in experiments that involve perturbation of cancer cells. Treating cancer cells with therapeutic agents or RNAi-mediating siRNA or shRNA molecules induces dramatic changes in the expression levels of many genes including commonly used reference genes. This phenomenon is due to (non-specific) off-target effects that are encountered upon delivery of such molecules [8], or indirect regulation after treatment. Therefore, we evaluated the expression of commonly used reference genes and expressed Alu repeats as internal controls for normalization in experiments that include perturbed cancer cell lines. Alu repeats are found in the untranslated regions of several thousands of known protein coding genes, and they have been reported to be useful as a single normalization factor for RT-qPCR reactions [9].

## Results

### Cancer Cell Perturbation Experiments

#### Treatment with nutlin-3

**Figure 1:**
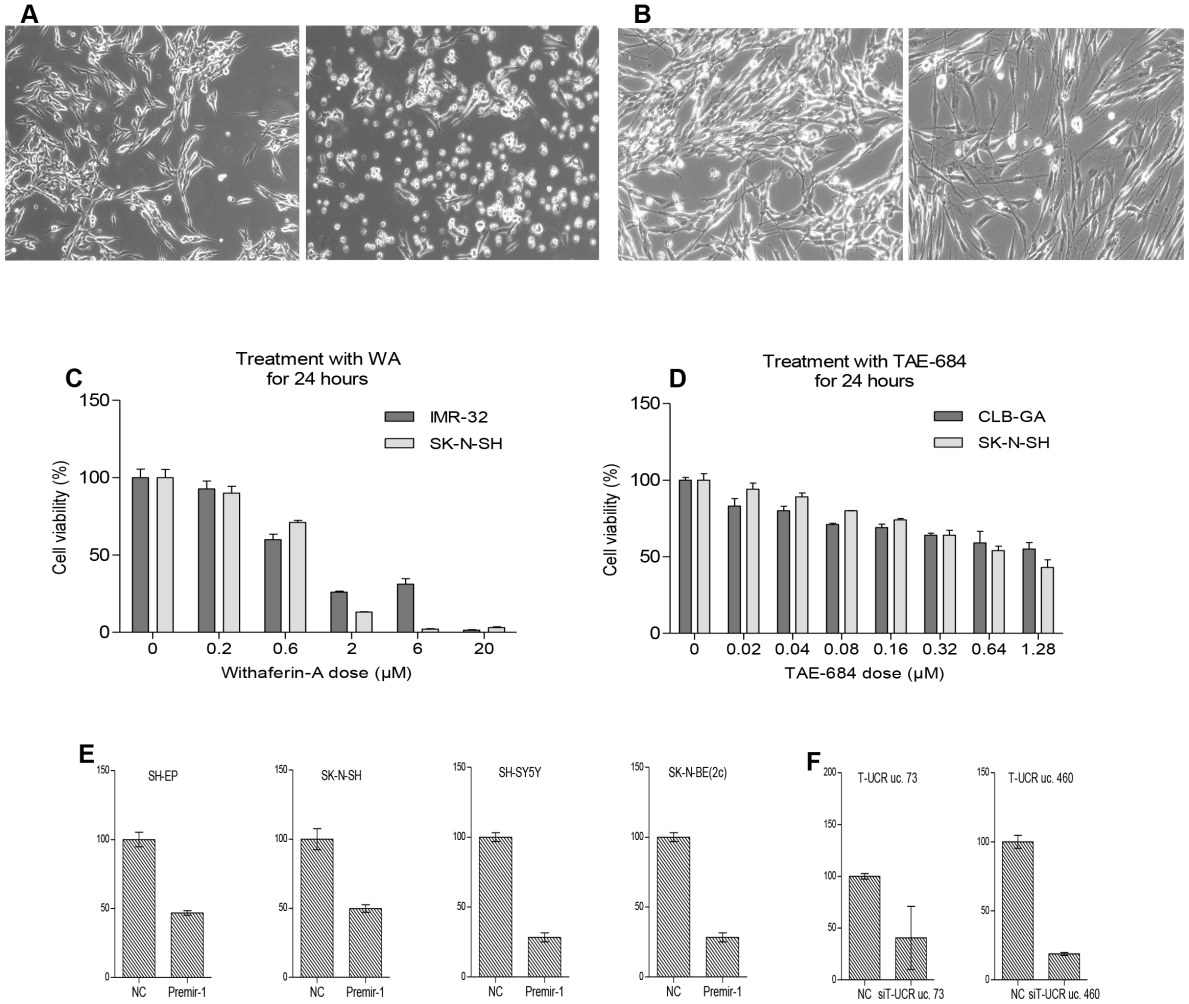
Various treatments of neuroblastoma cells. (A), nutlin-3 treated NGP cells, 16 µM (right) and vehicle control (left). (B), ATRA treated NGP cells with elongated neurites (right) and vehicle control (left). (C), cell viability assay of IMR-32 and SK-N-SH cells after treatment with increasing concentrations of withaferin-A for 24 hours. Error bars, SD (n = 3). (D), cell viability assay after treatment of CLB-GA and SK-N-SH cells with increasing concentrations of TAE-684 for 24 hours. Error bars, SD (n = 3). (E), RT-qPCR expression data of PTK9 in SH-EP, SK-N-SH, SH-SY5Y and SK-N-BE(2c) after transfection with miR-1 mimic or scrambled miRNA mimic serving as a negative control (NC). Error bars, SEM (n = 2). (F), RT-qPCR expression data of T-UCR uc. 73 (left) and T-UCR uc.460 (right) 24 hours after transfection of SH-EP cells with siRNA against T-UCR uc. 73 and siRNA against T-UCR uc. 460 respectively. Errors bars, SEM (n = 2).

Nutlin-3 is a small molecule that can specifically inhibit the p53-MDM2 interaction, which results in activation and stabilization of p53 [1], [2], [10]. Treatment with nutlin-3 induces apoptosis (Figure 1A), cell cycle arrest, differentiation, or senescence in neuroblastoma cells with wild-type *TP53* [2]–[6], [11].

#### Treatment with ATRA

All-*trans* retinoic acid (ATRA) is a small lipophilic molecule [7], [12] that inhibits proliferation and induces differentiation of neuroblastoma cells [4], [13]–[15]. We treated CLB-GA and NGP cells with 0 or 5 µM ATRA for one and five days, and observed that ATRA induces the outgrowth of neurites (Figure 1B).

#### Treatment with withaferin-A

Withaferin-A is a steroidal lactone purified from the medicinal plant *Withania somnifera*. This compound induces apoptosis in neuroblastoma cells and is an anti-angiogenic agent [8], [16]. We treated SK-N-SH and IMR-32 neuroblastoma cells with withaferin-A and observed reduced cell viability in a dose and time dependent manner (Figure 1C). We then treated SK-N-SH and IMR-32 cells with 0 or 1 µM withaferin-A for one day to evaluate the stability of the reference genes.

#### Treatment of neuroblastoma cell lines with TAE-684

TAE-684 is a small molecule inhibitor of activated anaplastic lymphoma kinase (ALK) [9], [17] and reduces cell viability of *ALK* mutated neuroblastoma cells [18]. After treating SK-N-SH and CLB-GA cells with TAE-684, we observed reduced cell viability in a dose and time dependent manner (Figure 1D). We then treated these 2 cell lines with 0, 0.1, 0.3 and 1 µM TAE-684 for 3, 6, 12, 24, and 48 hours to evaluate the stability of the reference genes.

#### Treatment of a NSCLC cell line with TAE-684

H3122 is a NSCLC cell line with an *EML4-ALK* fusion gene that was treated with TAE-684 in the same manner as described above.

#### Transient transfections of neuroblastoma cell lines with miR-1 mimic

MiR-1 targets the 3′-UTR of the *PTK9* mRNA leading to *PTK9* degradation [19]. MiR-1 is often used as a positive control in experiments with miRNA mimic transfections to evaluate target gene mRNA down regulation by qPCR. We performed transient transfections of SK-N-BE(2c), SK-N-SH, SH-EP, and SH-SY5Y neuroblastoma cells with miR-1 mimic, negative control (a scrambled miRNA mimic), or mock transfection for 24 hours (Figure 1E).

#### Transient transfections of SH-EP cells with siRNAs against transcribed ultraconserved regions

We transfected SH-EP cells with siRNA against both strands of T-UCR uc.460, and an siRNA against the negative strand of T-UCR uc.73.The knockdown efficiency is shown in Figure 1F. More details about the experimental design are found in File S1.

#### Transient transfection of leukemia cell lines with miR-223 mimic

MiR-223 was found to be highly expressed in T-cell acute lymphoblastic leukemia (T-ALL) [20]. However, three T-ALL cell lines, HPB-ALL, ALL-SIL and TALL-1 presented with a low miR-223 level. We expected that overexpression of oncogenic miR-223 in these cell lines would increase the proliferative capacity of the cells and prove the oncogenic potential of this miRNA (data not shown).

#### Optimization of concentration of *PHF6*-targeting siRNA in T-ALL cell lines

**Figure 2:**
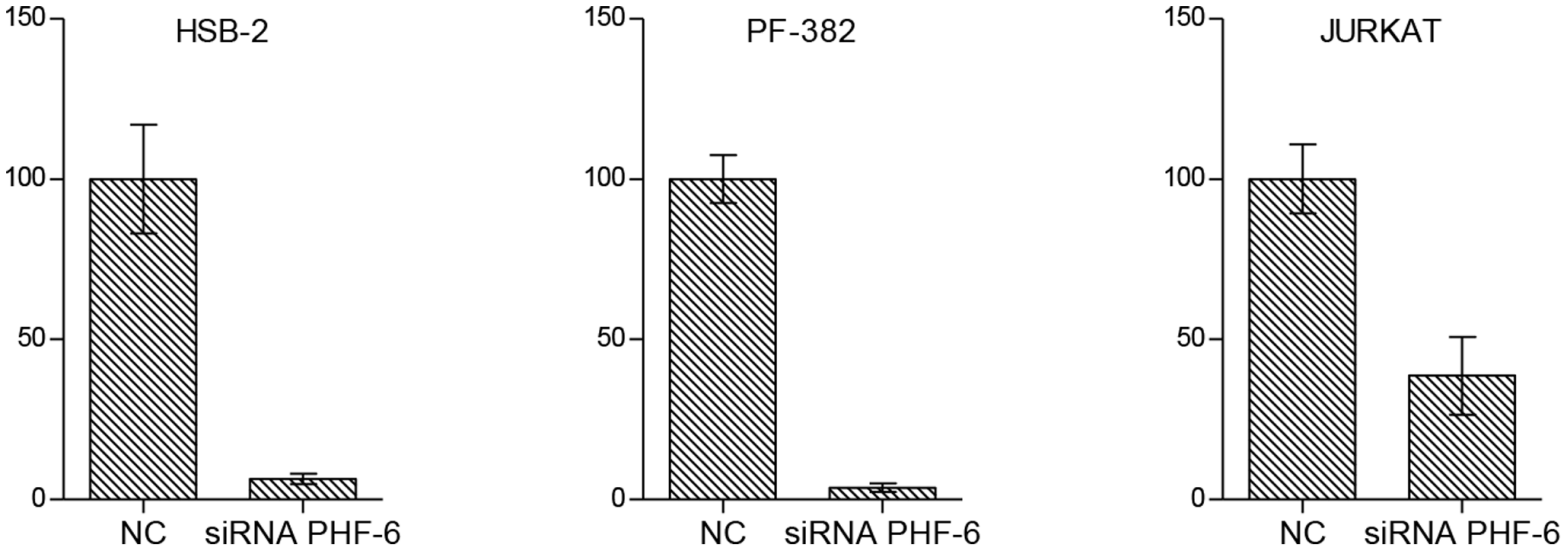
RT-qPCR expression data of *PHF6* in T-ALL cell line. JURKAT cell line after transfection of *PHF6*-targeting siRNA or scrambled siRNA (negative control: NC). Error bars, SEM (n = 2).

We transfected *PHF6* wild-type T-ALL cell line JURKAT and evaluated the knockdown efficiency on mRNA level (Figure 2). Next, we transiently transfected the HSB-2 and PF-382 T-ALL cell lines (both PHF6 wild-type) with PHF6-targeting siRNA. Significant PHF6 knock down was confirmed by qPCR (shown in Figure 2). More details about the experimental design are found in File S1.

#### Transient transfection of a melanoma cell line with siRNA against cyclophilin-B

WM9 is a melanoma cell line that was transiently transfected with 20 nM and 50 nM siRNA against cyclophilin-B (PPIB), or a scrambled negative control siRNA.

#### Treatment of breast cancer, AML, prostate cancer, colorectal cancer and neuroblastoma cell lines with JQ1

JQ1 is a small molecule compound that inhibits a bromodomain protein called BRD4. Targetting of this oncogene leads to growth inhibition of cancer cell lines. One known mechanism is through downregulation of MYCN. We treated two breast cancer cell lines (MCF-7 and SKBR3), one AML cell line (K562), one prostate cancer cell line (PC-3), one colorectal cancer cell line (SW-620) and one neuroblastoma cell line (SJNB-12) with 1 µM JQ1 for 24 and 48 hours.

### MCF-7 and HeLa Transcriptome PCR Arrays

We used commercially available ready-to-use cDNA plates from MCF7 (breast cancer cell line) and HeLa (cervical cancer cell line). Each cDNA sample has been synthesized from RNA extracted from a cell line that had been exposed to one of 90 different chemical inhibitors. These chemical inhibitors target a wide range of different pathways resulting in various perturbations of a two widely used cancer cell lines. The chemical inhibitors and the genes they target are listed in Table S1.

### Alu Repeats are the most Stably Expressed Reference Sequence

mRNA levels of 11 candidate reference assays were measured in all above described experiments and the average expression stability was calculated using the geNorm algorithm. GeNorm ranks the reference genes according to their stability value (referred to as the M-value) and calculates the optimal number of genes to be used for normalization in a given experiment using the V-value (Figure S1). The M-values can be used to rank the genes from the least to the most stable one [4].

**Figure 3:**
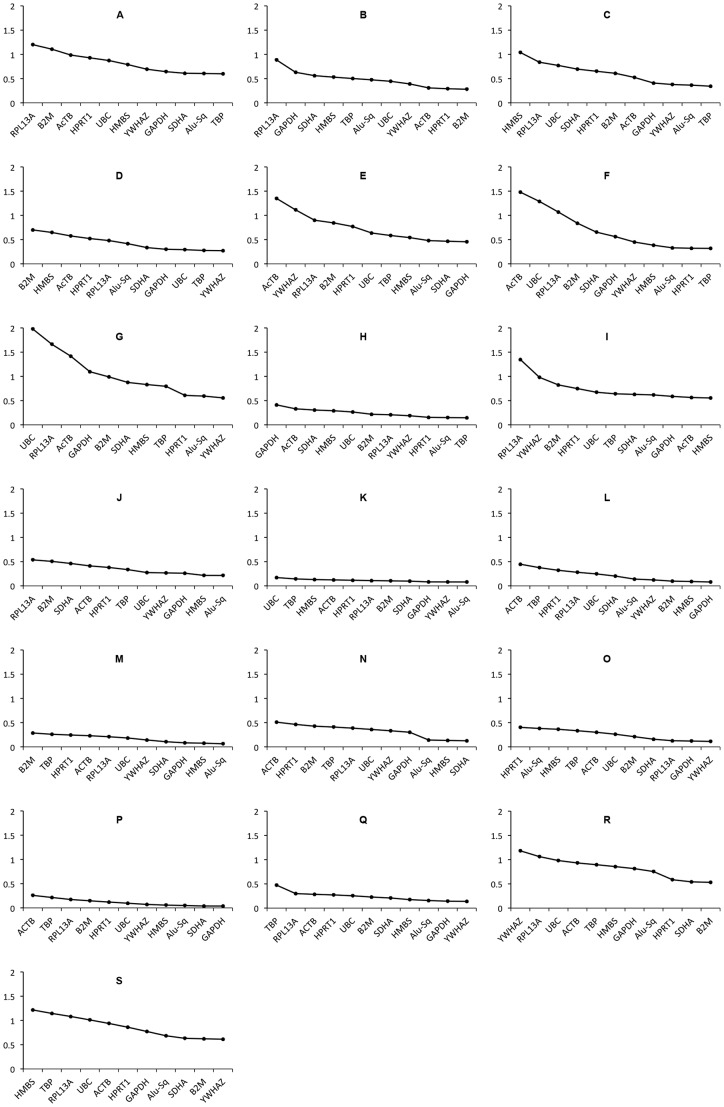
Average expression stability values of the reference genes. X-axis shows the ranking of reference genes, and Y-axis shows geNorm M-values. (A), nutlin-3 treated neuroblastoma cells. (B), ATRA treated neuroblastoma cells. (C), withaferin A treated neuroblastoma cells. (D), TAE-684 treated neuroblastoma cells. (E), neuroblastoma cells transfected with siRNAs against T-UCRs. (F), neuroblastoma cells transfected with miR-1 mimic. (G), T-ALL cell lines (HPB-ALL, ALL-SIL, and TALL-1) transfected with miR-223 mimic or negative control miR mimic. (H), T-ALL cell line JURKAT transfected with *PHF6*-targeting siRNA or negative control siRNA. (I), T-ALL cell lines (HSB-2 and PF-382) transfected with *PHF6*-targeting siRNA or negative control siRNA. (J), NSCL cell line (H3122) treated with crizotinib. (K), melanoma cell line (WM-9) transfected with siRNA against Cyclophilin-B. (L), AML cell line (K562) treated with JQ1. (M), breast cancer cell line (MCF-7) treated with JQ1. (N), breast cancer cell line (SKBR-3) treated with JQ1. (O), prostate cancer cell line (PC-3) treated with JQ1. (P), colorectal cell line (SW-620) treated with JQ1. (Q), neuroblastoma cell line (SJNB-12) treated with JQ1. (R), MCF-7 treated with 90 different chemical inhibitors. (S), cervical cancer cell line (HeLa) treated with 90 different chemical inhibitors.

In 13 of the 19 perturbation experiments performed, Alu repeats (Alu-Sq) were ranked among the three most stable reference assays (Figure 3). This observation prompted us to further analyze the potential value of Alu repeats as stable reference candidates.

**Figure 4:**
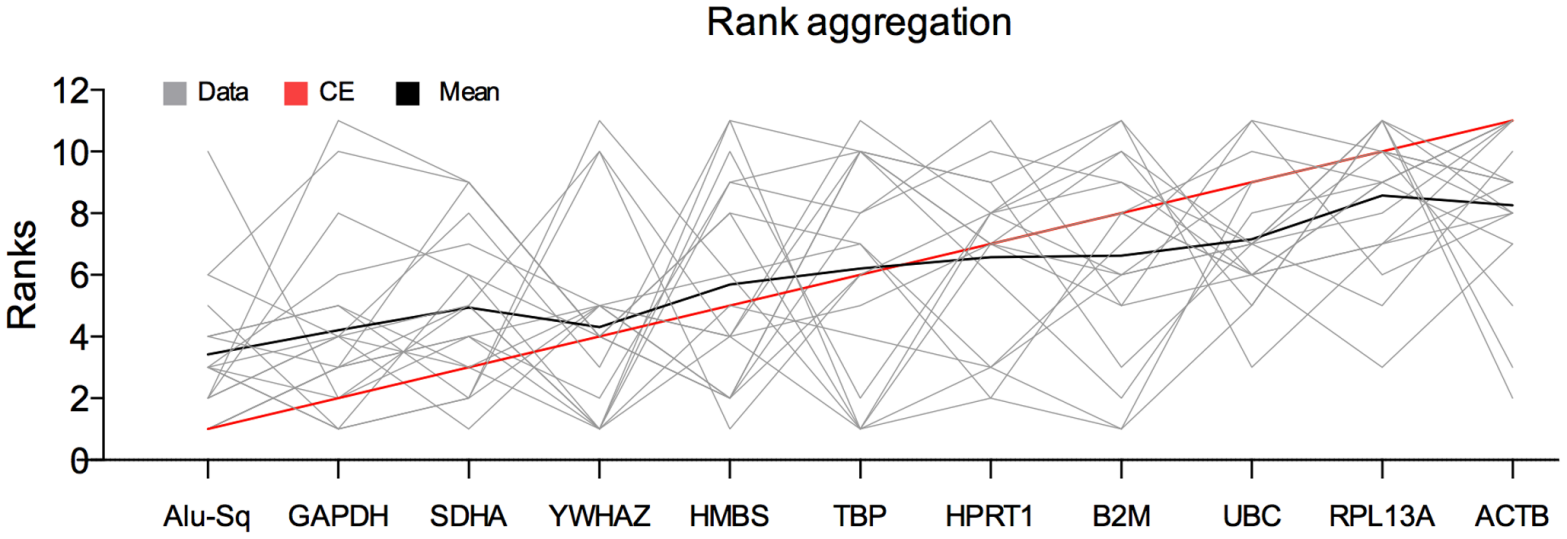
Rank aggregation analysis. Rank aggregation result using the cross entropy algorithm with spearman’s foot-rule weighted distance which shows a consensus order of reference genes from the most stable to the least stable.

A rank aggregation method based on voting theory (Borda count) was used to combine the 19 ranked lists of reference candidates [21]. This method tries to find an ordered list of reference assays as close as possible to all individual ordered lists by calculating the weighted Spearman’s footrule distance, and using a cross-entropy Monte Carlo algorithm or genetic algorithm [21]. The analysis of the 19 full gene lists, generated by the 19 different experiments, resulted in Alu repeats ranked at the first position (Figure 4). This confirmed that Alu repeats represent the most stable reference assay across all data sets.

## Discussion

RT-qPCR is the most commonly used method to quantify gene expression and accurate normalization is required to interpret the RT-qPCR data correctly. Normalization using endogenous control genes is a widely used method to correct for the technical variations that occur during RT-qPCR reactions. Until recently, a single non-validated reference gene has routinely been used as an internal control. We have previously reported that this strategy can lead to incorrect data with an error up to 3-fold in 25% of the cases [4]. Using multiple reference genes as internal controls for normalizing RT-qPCR data as well as using the appropriate reference genes for specific experimental purposes has already been strongly advocated in literature [2], [22]. Validating the stable expression of reference genes is an important issue in every single experimental procedure since cell manipulations such as treatment with therapeutic compounds can dramatically influence their expression.

In this study, we emphasize on the fact that proper selection of the reference genes is important for interpretation of RT-qPCR data and demonstrate that Alu repeats represent the most stable reference assay in a wide range of experimental conditions in eight different cancer types.

We selected 11 reference genes that are widely used in literature and that belong to different functional classes to avoid co-regulation. We selected structure related genes (*ACTB*, *RPL13A*), metabolism related genes (*HPRT*, *GAPDH*), and transcription related genes (*TBP*). The rest of the genes are not categorized specifically in one functional class. We also included expressed Alu repeats, which are abundantly interspersed throughout the genome. A commonly used normalization factor for RT-qPCR experiments in literature is 18S rRNA. The rRNA constitutes more than 90% of total RNA, and this fact led many researchers to use 18S rRNA as a control for normalization of gene expression data. However, it has been shown that equal fractions of rRNA do not necessarily ensure equal fractions of mRNA [23]. This concern is even of greater importance in cancer cell perturbation experiments as these perturbations may lead to differential expression of RNA polymerase I and/or II, or differential degradation of the two RNA populations [24], and consequently may result in further imbalances in the rRNA/mRNA ratio. In addition, the high amount of rRNA as compared to mRNA makes it difficult to subtract the background fluorescence in data analysis of RT-qPCR data [4]. Due to these reasons, using 18S rRNA as a control in RT-qPCR experiments could lead to false interpretation of gene expression data. We have therefore not evaluated 18S rRNA in our study.

RT-qPCR was performed for the 11 reference assays in 21 cancer cell lines derived from 9 different cancer entities using SYBR green technology. The cell lines were exposed to harsh treatment conditions, generating 19 different datasets with a total of 418 samples. The cells were treated with various chemical inhibitors including pro-apoptotic compounds and, differentiation-inducing agents, or transfected with miRNA mimics or siRNAs. Using geNorm [4] implemented in qbase+ [1], we calculated the stability value or M-value. The M-value is the average pairwise variation (standard deviation) of the log-transformed ratios of expression levels of paired candidate reference genes. This value not only allowed us to rank the reference genes in terms of their stability, but also to compare the stability of these reference genes across different experimental conditions. M-values and the rankings of the reference genes in all data sets are shown in Table 1. M-values lower than 0.5 are classified as good values [1]. Dispersion of M-values of every gene among all experiments are shown as a box plot in Figure S2.

**Table 1:** M-values for the candidate reference genes under different treatment conditions.

S	0.68	1.15	0.86	0.61	0.77	0.63	1.22	0.62	1.01	0.94	1.08
R	0.75	0.90	0.59	1.18	0.81	0.54	0.86	0.53	0.98	0.93	1.06
Q	0.16	0.47	0.27	0.14	0.14	0.21	0.18	0.23	0.25	0.28	0.30
P	0.05	0.22	0.12	0.07	0.04	0.04	0.06	0.15	0.10	0.26	0.17
O	0.38	0.33	0.40	0.11	0.12	0.16	0.36	0.21	0.26	0.30	0.13
N	0.14	0.41	0.46	0.33	0.30	0.13	0.13	0.43	0.36	0.51	0.39
M	0.06	0.26	0.25	0.14	0.08	0.11	0.08	0.29	0.18	0.23	0.21
L	0.14	0.38	0.32	0.12	0.08	0.20	0.09	0.10	0.25	0.45	0.28
K	0.08	0.14	0.12	0.08	0.08	0.10	0.13	0.10	0.17	0.12	0.11
J	0.22	0.34	0.38	0.27	0.26	0.46	0.22	0.51	0.28	0.41	0.54
I	0.62	0.64	0.75	0.98	0.59	0.63	0.55	0.82	0.67	0.57	1.35
H	0.15	0.15	0.16	0.19	0.41	0.31	0.29	0.22	0.27	0.33	0.21
G	0.59	0.80	0.61	0.56	1.10	0.88	0.83	0.99	1.98	1.42	1.66
F	0.33	0.32	0.32	0.45	0.56	0.65	0.39	0.84	1.29	1.48	1.07
E	0.48	0.58	0.77	1.11	0.46	0.47	0.54	0.85	0.64	1.35	0.90
D	0.42	0.28	0.52	0.27	0.30	0.34	0.65	0.70	0.29	0.58	0.48
C	0.37	0.34	0.65	0.38	0.41	0.70	1.04	0.61	0.77	0.52	0.84
B	0.47	0.50	0.29	0.39	0.63	0.56	0.53	0.28	0.45	0.31	0.88
A	0.61	0.60	0.93	0.69	0.64	0.61	0.79	1.11	0.87	0.99	1.20
	Alu-Sq	TBP	HPRT1	YWHAZ	GAPDH	SDHA	HMBS	B2M	UBC	ACTB	RPL13A

This table shows the 19 ordered lists of 11 reference candidates with their corresponding M-values. These M-values were used to rank the 11 reference candidates within each ordered list (in columns).

(**A**), nutlin-3 treated neuroblastoma cells. (**B**), ATRA treated neuroblastoma cells. (**C**), withaferin A treated neuroblastoma cells. (**D**), TAE-684 treated neuroblastoma cells. (**E**), neuroblastoma cells transfected with siRNAs against T-UCRs. (**F**), neuroblastoma cells transfected with miR-1 mimic. (**G**), T-ALL cell lines (HPB-ALL, ALL-SIL, and TALL-1) transfected with miR-223 mimic or negative control miRNA mimic. **(H**), T-ALL cell line JURKAT transfected with *PHF6*-targeting siRNA or negative control siRNA. (**I**), T-ALL cell lines (HSB-2 and PF-382) transfected with *PHF6*-targeting siRNA or negative control siRNA. (**J**), NSCL cell line (H3122) treated with crizotinib. (**K**), melanoma cell line (WM-9) transfected with siRNA against cyclophilin-B. (**L**), AML cell line (K562) treated with JQ1. (**M**), breast cancer cell line (MCF-7) treated with JQ1. (**N**), breast cancer cell line (SKBR-3) treated with JQ1. (**O**), prostate cancer cell line (PC-3) treated with JQ1. (**P**), colorectal cancer cell line (SW-620) treated with JQ1. (**Q**), neuroblastoma cell line (SJNB-12) treated with JQ1. (**R**), MCF-7 cells treated with 90 different chemical inhibitors. (**S**), cervical cancer cell line (HeLa) treated with 90 different chemical inhibitors.

After calculating the M-values and ranking the reference genes, we noticed that Alu repeats are ranked among the most stable reference genes in the vast majority of the datasets and generally have low M-values. We then applied a rank aggregation strategy [3] to determine the optimal ranking of the reference genes across all 19 data sets. This analysis confirmed that Alu repeats represent the most stable reference assay with acceptable M-values. All our gene expression measurements were done using SYBR green. Different technologies are currently available, and several studies have performed comparisons between the different platforms used [25]. Results have shown that SYBR green is in very good concordance with the widely used TaqMan gene expression assays. In the current study, we have used cell lines that give a good yield of RNA and perfect quality (tested using the Experion system from Bio-Rad). We believe that using such high quality RNA material and such abundant reference genes will generate reproducible results regardless of the platform being used**.** Our results strongly emphasize the importance of proper selection of reference genes for different experimental setups. In addition, we showed that Alu repeats can serve as a stable reference in most of the experimental conditions.

### Conclusions

The reliability of RT-qPCR data is based on the accurate normalization of the generated data using internal reference genes. The stability and suitability of putative endogenous control genes is a necessity for accurate normalization and for correct interpretation of gene expression data. In this study, we report that, among 11 commonly used reference genes, Alu repeats are the most stable reference sequence in cell lines from 9 different cancer types that were subjected to different perturbation experiments. We therefore recommend to include Alu repeats as a first candidate for normalization of RT-qPCR data.

## Materials and Methods

### Selection of the Reference Genes

The selection of the internal control genes evaluated in this study (Table 1) is based on a previous study published by our group [2], [4]–[6]. These genes are commonly used as reference genes in literature and belong to different pathways to avoid co-regulation of these genes upon different treatment conditions. We expanded this selection to include expressed Alu repeats.

### Cell Lines and Culturing of Cells

The cells used are established cell lines from 9 tumour types, neuroblastoma, T-ALL, melanoma, breast cancer, acute myeloid leukemia, prostate cancer, colorectal cancer, non-small-cell lung cancer, and cervical cancer. Details about culturing of the cell lines, and their source are mentioned in File S2.

### Drug Treatment and Cell Viability Assessment

Cells were treated with 16 µM nutlin-3 (Cayman Chemical, USA) or vehicle control (ethanol) for 1 and 5 days, 16 µM ATRA (Sigma-Aldrich, Belgium) or vehicle control (DMSO), 1 µM withaferin-A or vehicle control (ethanol), 0.1 µM, 0.3 µM or 1 µM TAE-684 (Novartis, Switzerland) or vehicle control (DMSO), 1 µM JQ1 (Cayman Chemical, USA) or vehicle control (DMSO) for the aforementioned time points. Cell viability was measured using CellTiter-Glo (Promega, Belgium) a luminscent ATP-based assay.

### Transfection with siRNAs and miRNA Mimics

SK-N-BE(2c), SK-N-SH, SH-EP, and SH-SY5Y cells were transfected with a miR-1 mimic using Dharmafect-2 (Dharmacon, United Kingdom) according to the manufacturer’s instructions. In short, 500.000 cells were seeded in 6-well plates in antibiotics free medium and supplied with 10% fetal calf serum. 24 hours later, cells were transfected using a final concentration of 100 nM of miR-1 mimic and 0.2% of Dharmafect-2. The cells were then harvested after 48 hours.

SH-EP cells were transfected with siRNAs against T-UCRs (uc.73 and uc.460) using Dharmafect-2 according to the instructions of the manufacturer. In short, 100.000 cells were cultured as described above and transfected using a final concentration of 50 nM siRNAs and 0.2% Dharmafect-2. The cells were harvested after 15, 24, 48 and 72 hours.

The T-ALL cell lines were electroporated at 250 V and 1000 µF (exponential decay pulse) (Genepulser II; Bio-Rad, Hercules, CA, USA) with 400 nM, 100 nM, 25 nM and 10 nM of *PHF6-*targeting siRNA (ON-TARGETplus SMARTpool; Dharmacon, Lafayette, CO, USA) for JURKAT cells and with 400 nM *PHF6-*targeting siRNA for HSB-2 and PF-382. ALL-SIL, T-ALL-1, and HPB-ALL were electroporated with 600 nM of miR-223 mimic. As a control, we electroporated the cells with similar concentrations of a scrambled siRNA (ON-TARGETplus Non-targeting Pool; Dharmacon, Lafayette, CO, USA) or without any siRNA. We harvested HSB- 2, PF-382, ALL-SIL, T-ALL-1, and HPB-ALL cells 1, 24, 48, 72, and 96 hours post-electroporation and JURKAT cells after 48 hours.

WM-9 cells were transfected with 20 nM, and 50 nM of siRNA pool against PPIB or scrambled siRNA (ON-TARGETplus Non-targeting Pool; Dharmacon, Lafayette, CO, USA).

The list of the chemical inhibitors used by Qiagen to treat MCF-7 and HeLa cells and their list of target genes are listed in Table S1.

### RT-qPCR

Extraction of total RNA, DNase treatment, cDNA synthesis, and SYBR Green I RT-qPCR from perturbed cells were carried out as described previously [4], [7]. The ready-to-use cDNA plates of MCF-7 and HeLa were ordered from Qiagen, Netherlands. The following primer sequences are available in the RTPrimerDB database (http://www.rtprimerdb.org) [4], [26]: ACTB (RTPrimerDB ID #1), B2M (#2), GAPDH (#3), HMBS (#4), HPRT1 (#5), RPL13A (#6), SDHA (#7), UBC(#8), YWHAZ (#9). The sequence of the Alu-Sq repeats primers are CATGGTGAAACCCCGTCTCTA for the forward primer and GCCTCAGCCTCCCGAGTAG for the reverse primer. The primer sequences of the TBP primers were as described [8], [27]. RNA quality index (RQI >8) was assessed for 20 samples selected at random using Experion (software version 3.2, Bio-Rad, Nazareth Eke, Belgium).

### Statistical Measurements and Data Analysis

GeNorm available in qbase+ (Biogazelle, http://www.qbaseplus.com) was used to calculate the M-values and quantify gene expression data. GeNorm is an algorithm that calculates a gene expression stability measure (M-value) of the selected reference genes. This is done by calculating the pairwise variation (standard deviation of logarithmically transformed expression ratios) of each reference gene with all other reference genes. The lowest M-value indicates the gene with the highest expression stability. Stepwise exclusion of the gene with the highest M-value allows the ranking of genes in terms of expression stability. The analysis of all individual ranked gene lists was done using the rank aggregation R package called “RankAggreg” [21]. RankAggreg was used to determine the most stable reference gene across all experiments. Rankaggreg is an R package for rank aggregation analysis. We used this package to analyze the individual ranked gene lists. These gene lists were ranked in terms of their M-values and the rank aggregation analysis allowed us to find the closest possible list to all individual lists generated by the individual experiments. More specifically, we used the Cross-Entropy (CE) Monte Carlo algorithm implemented in this package which starts by generating random lists and then converges towards the best optimal list through an iteration procedure that uses a distance function. Weighted Spearman’s footrule distance is used for this purpose and in our case the weight used is the M-value generated by the GeNorm algorithm for every gene in the individual lists.

## Supporting Information

Figure S1 *GeNorm V-values of the individual experiments* **.** GeNorm V-value is used to determine the optimal number of reference genes. GeNorm calculates the pairwise variation between 2 sequential normalization factors (NFs). The normalization factor is the geometric mean of expression of the selected reference genes. The normalization factor NF_n+1_ is the geometric mean of NF_n_ plus an additional reference gene. V_2/3_ is the variation between the NF_2_ and NF_3,_ and so on. Vandesompele *et al.* [4] proposed 0.15 as a cutoff below which there is no need to include an additional reference gene. (**A**), nutlin-3 treated neuroblastoma cells. (**B**), ATRA treated neuroblastoma cells. (**C**), withaferin A treated neuroblastoma cells. (**D**), TAE-684 treated neuroblastoma cells. (**E**), neuroblastoma cells transfected with siRNAs against T-UCRs. (**F**), neuroblastoma cells transfected with premiR-1. (**G**), T-ALL cell lines (HPB-ALL, ALL-SIL, and TALL-1) transfected with premiR-223 or negative control premiR. **(H**), T-ALL cell line JURKAT transfected with *PHF6*-targeting siRNA or negative control siRNA. (**I**), T-ALL cell lines (HSB-2 and PF-382) transfected with *PHF6*-targeting siRNA or negative control siRNA. (**J**), NSCL cell line (H3122) cells treated with crizotinib. (**K**), melanoma cell line (WM-9) transfected with siRNA against cyclophilin-B. (**L**), AML cell line (K562) treated with JQ1. (**M**), breast cancer cell line (MCF-7) treated with JQ1. (**N**), breast cancer cell line (SKRB-3) treated with JQ1. (**O**), prostate cancer cell line (PC-3) treated with JQ1. (**P**), colorectal cell line (SW-620) treated with JQ1. (**Q**), neuroblastoma cell line (SJNB-12) treated with JQ1. (**R**), MCF-7 cells treated with 90 different chemical inhibitors. (**S**), cervical cancer cell line (HeLa) treated with 90 different chemical inhibitors.
(TIF)

Figure S2 *Dispersion of M-values*. Box plot showing the dispersion of the M-values of every reference genes across al datasets.
(TIF)

Table S1 Chemical inhibitors. A list of the 90 chemical inhibitors and the genes they target.
(XLSX)

File S1 *Experimental design and manipulation of the cell lines.* Transient transfections of SH-EP cells with siRNAs against transcribed ultraconserved regions, and optimization of concentration of *PHF-6*-targetting siRNAs in T-ALL cell lines.
(DOCX)

File S2 *Cell lines and culturing of cells*. Information about the source and the culturing conditions of the cell lines.
(DOCX)
