# Supplementary figures and images for: Effective Alu Repeat Based RT-Qpcr Normalization in Cancer Cell Perturbation Experiments
Source: PLoS One. 2013 Aug 14;8(8):e71776. doi: 10.1371/journal.pone.0071776 (PMC3743747; doi:10.1371/journal.pone.0071776)

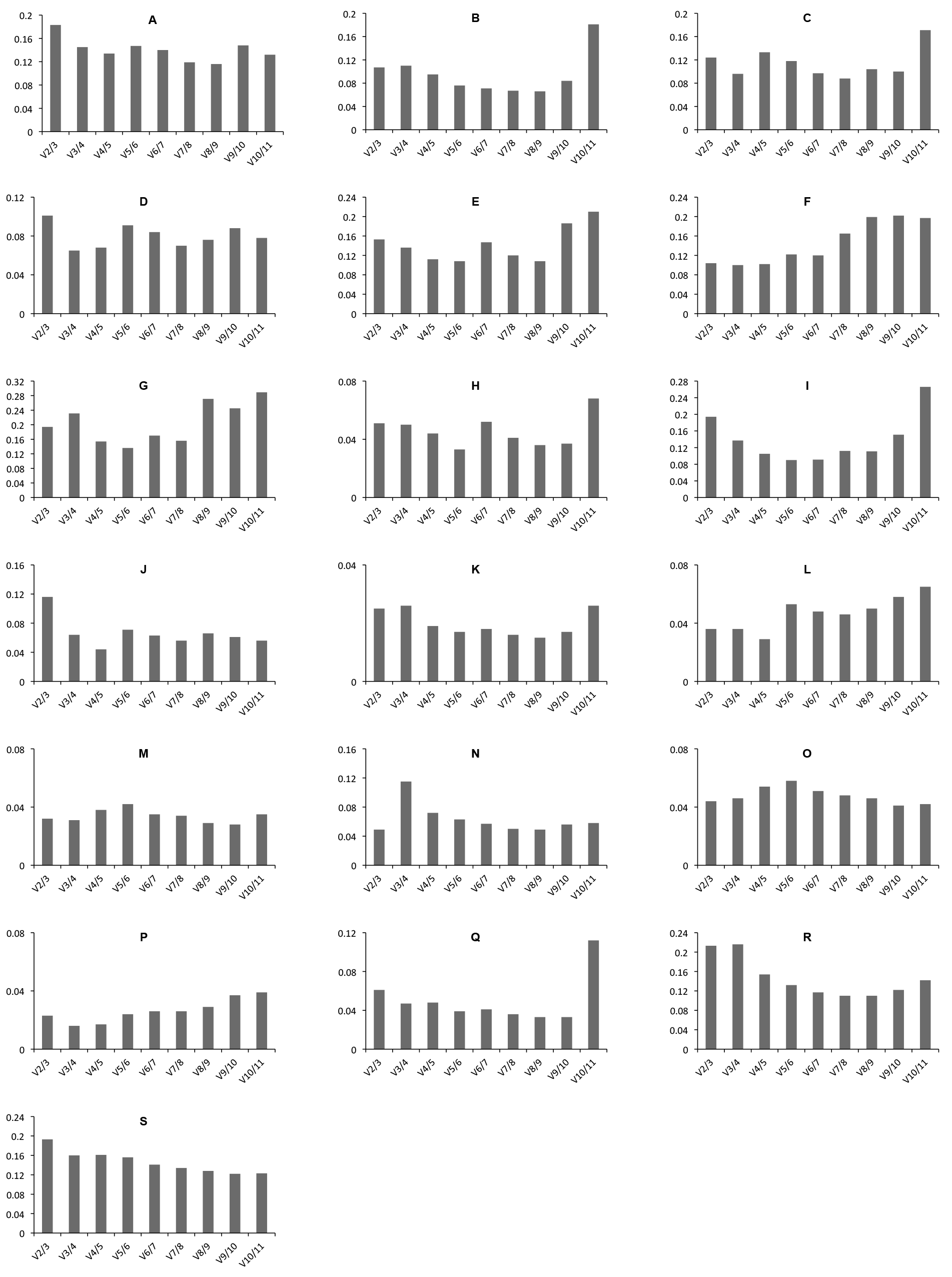

Supplement: Figure S1 — GeNorm V-values of the individual experiments . GeNorm V-value is used to determine the optimal number of reference genes. GeNorm calculates the pairwise variation between 2 sequential normalization factors (NFs). The normalization factor is the geometric mean of expression of the selected reference genes. The normalization factor NFn+1 is the geometric mean of NFn plus an additional reference gene. V2/3 is the variation between the NF2 and NF3, and so on. Vandesompele et al. [4] proposed 0.15 as a cutoff below which there is no need to include an additional reference gene. (A), nutlin-3 treated neuroblastoma cells. (B), ATRA treated neuroblastoma cells. (C), withaferin A treated neuroblastoma cells. (D), TAE-684 treated neuroblastoma cells. (E), neuroblastoma cells transfected with siRNAs against T-UCRs. (F), neuroblastoma cells transfected with premiR-1. (G), T-ALL cell lines (HPB-ALL, ALL-SIL, and TALL-1) transfected with premiR-223 or negative control premiR. (H), T-ALL cell line JURKAT transfected with PHF6-targeting siRNA or negative control siRNA. (I), T-ALL cell lines (HSB-2 and PF-382) transfected with PHF6-targeting siRNA or negative control siRNA. (J), NSCL cell line (H3122) cells treated with crizotinib. (K), melanoma cell line (WM-9) transfected with siRNA against cyclophilin-B. (L), AML cell line (K562) treated with JQ1. (M), breast cancer cell line (MCF-7) treated with JQ1. (N), breast cancer cell line (SKRB-3) treated with JQ1. (O), prostate cancer cell line (PC-3) treated with JQ1. (P), colorectal cell line (SW-620) treated with JQ1. (Q), neuroblastoma cell line (SJNB-12) treated with JQ1. (R), MCF-7 cells treated with 90 different chemical inhibitors. (S), cervical cancer cell line (HeLa) treated with 90 different chemical inhibitors. (TIF) [file pone.0071776.s001.tif]
