# Supplementary material for: Effective Alu Repeat Based RT-Qpcr Normalization in Cancer Cell Perturbation Experiments
Source: PLoS One. 2013 Aug 14;8(8):e71776. doi: 10.1371/journal.pone.0071776 (PMC3743747; doi:10.1371/journal.pone.0071776)
Supplement: File S1 — Experimental design and manipulation of the cell lines. Transient transfections of SH-EP cells with siRNAs against transcribed ultraconserved regions, and optimization of concentration of PHF-6-targetting siRNAs in T-ALL cell lines. (DOCX) [file pone.0071776.s004.docx]

## Experimental design and manipulation of the cell lines

## Transient transfections of SH-EP cells with siRNAs against transcribed ultraconserved regions

Recently, a new class of non-coding RNAs called transcribed ultraconserved regions (T-UCRs) was shown to be implicated in tumorigenesis. For example, T-UCR uc.73 was described to play an essential role during apoptosis [1]. We showed previously that T-UCR expression is also deregulated in neuroblastoma cells and that T-UCRs are implicated in a wide range of cellular functions [2]. Our study showed that two T-UCRs (uc.460 and uc.279) are significantly upregulated in *MYCN*-amplified (MNA) tumors. We considered uc.460 for further functional work because it was most highly upregulated.

**Optimization of concentration of *PHF6*-targeting siRNA in T-ALL cell lines**

*PHF6* was identified as a novel X-linked tumor suppressor gene in T-ALL. Inactivating mutations and deletions in this gene were identified in ~38% of adult and ~16% of pediatric primary T-ALL samples [3]. To evaluate the role of *PHF6* in T-ALL, we performed transient knockdown experiments in *PHF6* wild-type T-ALL cell lines by use of electroporation. To achieve this goal, we optimized the concentration of *PHF6*-targeting siRNA in the *PHF6* wild-type T-ALL cell line JURKAT and evaluated the knockdown efficiency on mRNA level

1. Calin GA, Liu C-G, Ferracin M, Hyslop T, Spizzo R, Sevignani C, Fabbri M, Cimmino A, Lee EJ, Wojcik SE, Shimizu M, Tili E, Rossi S, Taccioli C, Pichiorri F, Liu X, Zupo S, Herlea V, Gramantieri L, Lanza G, Alder H, Rassenti L, Volinia S, Schmittgen TD, Kipps TJ, Negrini M, Croce CM: **Ultraconserved regions encoding ncRNAs are altered in human leukemias and carcinomas.** *Cancer Cell* 2007, **12**:215–229.

2. Mestdagh P, Fredlund E, Pattyn F, Rihani A, Van Maerken T, Vermeulen J, Kumps C, Menten B, De Preter K, Schramm A, Schulte J, Noguera R, Schleiermacher G, Janoueix-Lerosey I, Laureys G, Powel R, Nittner D, Marine J-C, Ringnér M, Speleman F, Vandesompele J: **An integrative genomics screen uncovers ncRNA T-UCR functions in neuroblastoma tumours.** *Oncogene* 2010, **29**:3583–3592.

3. Van Vlierberghe P, Palomero T, Khiabanian H, Van Der Meulen J, Castillo M, Van Roy N, De Moerloose B, Philippé J, González-García S, Toribio ML, Taghon T, Zuurbier L, Cauwelier B, Harrison CJ, Schwab C, Pisecker M, Strehl S, Langerak AW, Gecz J, Sonneveld E, Pieters R, Paietta E, Rowe JM, Wiernik PH, Benoit Y, Soulier J, Poppe B, Yao X, Cordon-Cardo C, Meijerink J, Rabadan R, Speleman F, Ferrando A: **PHF6 mutations in T-cell acute lymphoblastic leukemia.** *Nat Genet* 2010, **42**:338–342.
