# Supplementary material for: Effective Alu Repeat Based RT-Qpcr Normalization in Cancer Cell Perturbation Experiments
Source: PLoS One. 2013 Aug 14;8(8):e71776. doi: 10.1371/journal.pone.0071776 (PMC3743747; doi:10.1371/journal.pone.0071776)
Supplement: File S2 — Cell lines and culturing of cells. Information about the source and the culturing conditions of the cell lines. (DOCX) [file pone.0071776.s005.docx]

| Cell line | Source | Reference |
| --- | --- | --- |
| \| CLBGA \| \| --- \| | V Combaret** | [1] |
| IMR-32 | R Versteeg* | [2] |
| NGP | R Versteeg | [3] |
| SK-N-SH | R Versteeg | [4] |
| SHEP | R Versteeg | [5] |
| SK-N-BE(2c) | R Versteeg | [6] |
| SH-SY5Y | R Versteeg | [7] |
| SJNB-12 | R Versteeg | [8] |
| HSB-2 | DSMZ-ACC 435 | [9] |
| PF-382 | DSMZ-ACC 38 | [10] |
| JURKAT | DSMZ-**ACC 282** | [11] |
| HPB-ALL | DSMZ-**ACC 483** | [12] |
| ALL-SIL | DSMZ-**ACC 511** | [13] |
| TALL-1 | DSMZ-**ACC 521** | [14] |
| MCF-7 | Qiagen | [15] |
| SW-620 | Marc M Mareel❖ | [16] |
| PC-3 | Marc Bracke§ | [17] |
| SKBR-3 | De Potter∞ | [18] |
| HeLa | Qiagen | [19] |

## Cell lines and culturing of cells

The cells used are established cell lines from two tumour types, neuroblastoma and T-ALL. The cell lines used in this study are: neuroblastoma cell lines (NGP, SK-N-SH, IMR-32, CLB-GA, SHEP, SK-N-BE(2c), SH-SY5Y, and SJNB-12). T-ALL cell lines (HSB-2, PF-382, JURKAT, HPB-ALL, ALL-SIL, and TALL-1). Breast cancer cell lines (MCF-7, and SKBR3), melanoma cell line (WM-9), acute myeloid leukemia cell line (AML), prostate cancer cell line (PC-3), colorectal cancer cell line (SW-620), non-small-cell lung cancer cell line (H3122), and cervical cancer cell line (HeLa).

All cell lines were cultured in T25 flasks in RPMI- 1640 medium supplemented with 10% fetal calf serum, 2 mM glutamine, 100 IU/mL penicillin, and 100 μg/mL streptomycin (medium and supplements from Gibco–Invitrogen, Belgium) in a humidified atmosphere with 5% CO_2_ at 37 °C.

The source of the cell lines is summarized in the following table:

* Rogier Versteeg (Department of Human Genetics, Amsterdam, The Netherlands)

** Centre Léon Bérard, Laboratoire de Recherche Translationnelle, Lyon, France.

§ Department of Medical Protein Research, VIB, B-9000 Ghent, Belgium.

∞ Christian R. De Potter, M.D., N. Goormaghtigh Institute for Pathology, De Pintelaan 185, B-9000 Gent, Belgium.

❖Laboratory of Experimental Cancerology, Department of Radiotherapy and Nuclear Medicine, Ghent University Hospital, B-9000 Ghent, Belgium

1. Combaret V, Turc-Carel C, Thiesse P, Rebillard AC, Frappaz D, Haus O, Philip T, Favrot MC: **Sensitive detection of numerical and structural aberrations of chromosome 1 in neuroblastoma by interphase fluorescence in situ hybridization. Comparison with restriction fragment length polymorphism and conventional cytogenetic analyses.** *Int. J. Cancer* 1995, **61**:185–191.

2. el-Deiry WS, Kern SE, Pietenpol JA, Kinzler KW, Vogelstein B: **Definition of a consensus binding site for p53.** *Nat Genet* 1992, **1**:45–49.

3. Brodeur GM, Goldstein MN: **Histochemical demonstration of an increase in acetylcholinesterase in established lines of human and mouse neuroblastomas by nerve growth factor.** *Cytobios* 1976, **16**:133–138.

4. Biedler JL, Helson L, Spengler BA: **Morphology and growth, tumorigenicity, and cytogenetics of human neuroblastoma cells in continuous culture.** *Cancer Res.* 1973, **33**:2643–2652.

5. Baker DL, Reddy UR, Pleasure D, Thorpe CL, Evans AE, Cohen PS, Ross AH: **Analysis of nerve growth factor receptor expression in human neuroblastoma and neuroepithelioma cell lines.** *Cancer Res.* 1989, **49**:4142–4146.

6. Biedler JL, Roffler-Tarlov S, Schachner M, Freedman LS: **Multiple neurotransmitter synthesis by human neuroblastoma cell lines and clones.** *Cancer Res.* 1978, **38**:3751–3757.

7. Ross RA, Spengler BA, Biedler JL: **Coordinate morphological and biochemical interconversion of human neuroblastoma cells.** *J. Natl. Cancer Inst.* 1983, **71**:741–747.

8. Johnson MR, Look AT, DeClue JE, Valentine MB, Lowy DR: **Inactivation of the NF1 gene in human melanoma and neuroblastoma cell lines without impaired regulation of GTP.Ras.** *Proc. Natl. Acad. Sci. U.S.A.* 1993, **90**:5539–5543.

9. Adams RA, Flowers A, Davis BJ: **Direct implantation and serial transplantation of human acute lymphoblastic leukemia in hamsters, SB-2.** *Cancer Res.* 1968, **28**:1121–1125.

10. Pegoraro L, Fierro MT, Lusso P, Giovinazzo B, Lanino E, Giovarelli M, Matera L, Foa R: **A novel leukemia T-cell line (PF-382) with phenotypic and functional features of suppressor lymphocytes.** *J. Natl. Cancer Inst.* 1985, **75**:285–290.

11. Schneider U, Schwenk HU, Bornkamm G: **Characterization of EBV-genome negative "null" and “T” cell lines derived from children with acute lymphoblastic leukemia and leukemic transformed non-Hodgkin lymphoma.** *Int. J. Cancer* 1977, **19**:621–626.

12. Morikawa S, Tatsumi E, Baba M, Harada T, Yasuhira K: **Two E-rosette-forming lymphoid cell lines.** *Int. J. Cancer* 1978, **21**:166–170.

13. Graux C, Cools J, Melotte C, Quentmeier H, Ferrando A, Levine R, Vermeesch JR, Stul M, Dutta B, Boeckx N, Bosly A, Heimann P, Uyttebroeck A, Mentens N, Somers R, MacLeod RAF, Drexler HG, Look AT, Gilliland DG, Michaux L, Vandenberghe P, Wlodarska I, Marynen P, Hagemeijer A: **Fusion of NUP214 to ABL1 on amplified episomes in T-cell acute lymphoblastic leukemia.** *Nat Genet* 2004, **36**:1084–1089.

14. Miyoshi I, Hiraki S, Tsubota T, Kubonishi I, Matsuda Y, Nakayama T, Kishimoto H, Kimura I, Masuji H: **Human B cell, T cell and null cell leukaemic cell lines derived from acute lymphoblastic leukaemias.** *Nature* 1977, **267**:843–844.

15. Kurebayashi J, Kurosumi M, Sonoo H: **A new human breast cancer cell line, KPL-1 secretes tumour-associated antigens and grows rapidly in female athymic nude mice.** *Br. J. Cancer* 1995, **71**:845–853.

16. Leibovitz A, Stinson JC, McCombs WB, McCoy CE, Mazur KC, Mabry ND: **Classification of human colorectal adenocarcinoma cell lines.** *Cancer Res.* 1976, **36**:4562–4569.

17. Kaighn ME, Narayan KS, Ohnuki Y, Lechner JF, Jones LW: **Establishment and characterization of a human prostatic carcinoma cell line (PC-3).** *Invest Urol* 1979, **17**:16–23.

18. Trempe GL: **Human breast cancer in culture.** *Recent Results Cancer Res.* 1976:33–41.

19. SCHERER WF, SYVERTON JT, GEY GO: **Studies on the propagation in vitro of poliomyelitis viruses. IV. Viral multiplication in a stable strain of human malignant epithelial cells (strain HeLa) derived from an epidermoid carcinoma of the cervix.** *J. Exp. Med.* 1953, **97**:695–710.
